# Towards standardized assessment of surgical difficulty in robotic hepatobiliary surgery: a comparative validation study

**DOI:** 10.1007/s11701-026-03472-9

**Published:** 2026-07-20

**Authors:** Simone Conci, Giovanni Catalano, Serena Di Paolo, Tommaso Campagnaro, Mario De Bellis, Laura Alaimo, Edoardo Poletto, Andrea Ruzzenente

**Affiliations:** Division of General and Hepatobiliary Surgery, Department of Surgical Sciences, Dentistry, Gynecology and Pediatrics, University of Verona, G.B. Rossi University Hospital, P. le L.A. Scuro 10, Verona, 37134 Italy

## Abstract

The advent of robotic platforms in hepatobiliary surgery has expanded the range of procedures amenable to a minimally invasive approach. Surgical difficulty assessment is essential for optimal patient selection, particularly during the surgeon’s learning curve. Although several difficulty scoring systems (DSS) have been proposed, most have been developed for laparoscopic liver surgery. Therefore, their predictive performance in robotic series remains uncertain. Patients undergoing robotic liver resection for various indications were identified from a prospectively-maintained database. The Tampa, IWATE, Halls, Hasegawa, and Kawaguchi DSS were calculated based on variables available at the time of surgery. The predictive accuracy of the five DSS when assessing intraoperative and postoperative outcomes were compared using the ROC-AUC. Among 187 patients, unplanned conversion occurred in 4.3% (*n* = 8), severe morbidity in 14.4% (*n* = 27) and 90-day mortality in 3.2% (*n* = 6). Textbook Outcome in Liver Laparoscopic Surgery (TOLLS) was achieved in 66.8% (*n* = 125). The categorization of patients into surgical difficulty categories varied drastically among the DSSs. The Tampa DSS demonstrated the highest discriminatory ability for predicting unplanned conversion (AUC = 0.735), severe complication (AUC = 0.743), 90-day mortality (AUC = 0.722), and TOLLS achievement (AUC = 0.702) (Fig. 1). In this robotic series, the Tampa DSS showed superior performance in predicting postoperative outcomes. Standardized assessment of surgical complexity is crucial for accurate patient selection, reliable benchmarking, and tailored implementation of learning curves.

**Supplementary Information:**

The online version contains supplementary material available at 10.1007/s11701-026-03472-9.

## Introduction

Over the last decades, minimally invasive surgery (MIS) has reshaped the standard of care for several abdominal procedures. In liver surgery, however, early adoption was constrained by safety concerns, technical complexity, demanding learning curves, and lack of appropriate equipment [1]. Since the first laparoscopic liver resection in the early 1990s, minimally invasive liver surgery (MILS) substantially evolved through technological innovation, growing surgical expertise, and advances in perioperative care [2, 3]. When technically feasible, minimally invasive approaches have demonstrated improved short-term outcomes while yielding comparable oncologic results compared with open surgery, thereby becoming the preferred approach in appropriately selected patients [4, 5]. The advent of the robotic platform represents the latest paradigm shift in MILS. Robotic-assisted liver resection provides enhanced 3-D and immersive view, articulated instruments, tremor filtration, and motion scaling, potentially expanding the boundaries of minimally invasive liver and biliary surgery [6–8]. These technological refinements may also facilitate complex procedures, such as biliary and vascular resection and reconstruction.

Despite increasing global adoption, minimally-invasive liver surgery (MILS) remains a challenging and highly operator-dependent procedure characterized by a difficult standardization of the technique and a steep learning curve. In this setting, accurate preoperative evaluation of technical difficulty and appropriate case selection are crucial, not only to optimize patient safety, but also to structure training programs and benchmark institutional performance. Therefore, many attempts have been made to develop accurate difficulty scoring systems (DSS) to standardize complexity assessment and predict perioperative risk. During the 2nd International Consensus Conference on Laparoscopic Liver Resection held in Morioka, Japan, a difficulty scoring system for selecting patients suitable for laparoscopic liver resection was created based on preoperative factors [9]. Similarly, other difficulty scoring systems such as the Kawaguchi, Halls, and Hasegawa DSS, have been mainly developed and tested predominantly on laparoscopic cohorts [10–13]. 

However, whether these laparoscopy-based DSS retain discriminatory capacity in the robotic setting remains uncertain. The absence of platform-specific validation represents a significant methodological gap, particularly as robotic liver surgery increasingly encompasses complex resections traditionally considered high difficulty. The recently proposed Tampa score was designed specifically for robotic liver and biliary surgery, yet comparative data evaluating its performance against established DSS are limited [14]. 

In this framework, this study aimed to critically assess and compare the stratification and predictive performance of the main difficulty scoring systems (i.e., Halls, Kawaguchi, IWATE, Hasegawa, and Tampa) in a cohort of patients undergoing robotic-assisted liver and biliary surgery. By identifying the best model to evaluate technical complexity and postoperative outcomes, we sought to clarify the role of existing DSS in robotic surgery and inform a more standardized framework for complexity stratification in robotic liver and biliary surgery.

## Methods

### Patient population and study variables

Patients who underwent robot-assisted liver resection for benign or malignant diseases at the Division of General and Hepatobiliary Surgery, University of Verona (Verona, Italy), between 2014 and 2025 were identified from a prospectively-maintained database. Patients younger than 18 years, as well as those with incomplete data required to calculate difficulty scores or assess perioperative outcomes, were excluded. Patient demographics and clinical characteristics included age, sex, body-mass index (BMI), ASA (American Society of Anesthesiology) class, adjusted Charlson Comorbidity index (CCI), underlying liver disease and the presence of clinical portal hypertension (i.e., enlarged spleen or esophageal varices on preoperative imaging or a preoperative platelet count ≤ 100.000/mm³), receipt of systemic therapy, and tumor characteristics such as lesions size and number and proximity to main vessels. Liver resections were classified as minor or major according to the Brisbane classification and the Southampton guidelines for laparoscopic liver surgery [15, 16]. Specifically, liver resections were defined “anatomically major” when involved three or more anatomical segments according to the Coinaud classification or “technically major” when included at least one posterosuperior or difficult-to-access segment (i.e., I, IVa, VII, or VIII). Operative time estimated blood loss (EBL), performance and duration of hilar clamping, unplanned conversion, and intraoperative adverse events (according to the Oslo classification) were extracted from the operative reports [17]. comment. All robotic liver resections were performed by AR, SC and TC as console surgeons, each with over ten years of experience and at least 200 liver resections as first surgeon in both open and laparoscopic approaches, who completed formal training on the da Vinci^®^ System (i.e., TR100 Table and Console surgeon). The institutional review board approved the study.

### Study outcomes

Intraoperative technical complexity was evaluated based on EBL, operative time, and intraoperative adverse events. Perioperative outcomes included intraoperative adverse events, unplanned conversion, postoperative complications, 90-day mortality, 30-day readmission, and reoperation. Postoperative complications were classified according to the Clavien-Dindo classification, and grade 3 or greater complication were defined as “severe”. Comprehensive surgical performance was evaluated using the Textbook Outcome in Liver Laparoscopic Surgery (TOLLS) composite metric, which was defined as the simultaneous fulfillment of six criteria: absence of intraoperative adverse events, absence of severe postoperative complications, no 90-day mortality or 30-day readmission, and achievement of a radical resection (i.e., R0) [18]. 

### Difficulty scoring systems

All DSS were calculated based on variables available at the time of surgery (**Table Supplement 1**). While the Tampa scoring system was calculated for all patients included in the study cohort, the IWATE, Halls, Hasegawa, and Kawaguchi scores were calculated only for patients undergoing parenchymal liver resection, thus excluding patients who underwent isolated biliary resection and/or reconstruction, as these scores have limited applicability in this setting. The Halls (i.e., Southampton) DSS divides patients into 4 categories (i.e., low, moderate, high, and extreme risk) based on the risk of developing intraoperative adverse events, and is based on: type of resection, tumor size and etiology, history of previous laparotomic liver surgery or neoadjuvant systemic therapy [11, 19]. The Hasegawa DSS was developed to predict operative time as a surrogate of surgical complexity and incorporates type of resection, tumor location, BMI, and platelet count, classifying patients into a low-, medium-, and high-difficulty categories [12]. The IWATE DSS represents an update of the original score proposed by Ban et al., which evaluates overall difficulty of liver surgery: while both DSS are based on tumor location and size and proximity to vessels, the IWATE DSS additionally incorporating Child-Pugh score, better distinction between different liver segments, and accounts for hand-assisted or hybrid approaches [9, 20]. The Kawaguchi (i.e., IMM, *Institut Mutualiste Montsouris*) DSS divides patients into three categories based on extension of liver resection, with group I including patients who undergo wedge resection or left lateral sectionectomy, whereas group III includes right hepatectomy as well as posterosuperior segmentectomy [10]. The Tampa DSS was specifically designed for robotic surgery and integrates preoperative variables such as BMI, receipt of neoadjuvant chemotherapy, tumor characteristics, as well as procedural variables such as lymphadenectomy and the need for biliary resection and/or reconstruction, with each patient receiving a weighted score from 1 to 49 and thus differentiating the surgical procedures into 4 groups of increasing technical complexity (“less demanding”, “intermediate”, “more demanding”, and “most demanding”) [14]. 

### Statistical analysis

Continuous variables are reported as median and interquartile range (IQR) and were compared using the Wilcoxon rank sum test. Categorical variables are presented as frequencies and percentages (%) and were compared using the Chi-square test or Fisher’s exact test, as appropriate. The discriminatory performance of each DSS was evaluated using the area under the receiver operating characteristic curve (AUC). Multivariable logistic regression models based on each different DSS were developed for the outcomes of interest using selected patient covariates (i.e., age, sex, BMI, comorbidities, liver function) to evaluate goodness-of-fit, discrimination, and clinical utility. Goodness-of-fit was assessed using the Akaike Information Criterion (AIC), with lower values indicating a superior balance between model accuracy and complexity. Model discrimination was evaluated using the concordance index (C-index). Decision Curve Analysis (DCA) was utilized to compare the true clinical value of the different models across a continuum of threshold probabilities. Multivariable logistic regression analysis was used to assess the association of the DSS with selected clinical outcomes, and results were reported as odds ratios (OR) with 95% confidence intervals (CI). A two-sided P-value < 0.05 was considered statistically significant. All statistical analyses were performed using IBM SPSS Statistics 27 (IBM Corp., Armonk, NY, USA).

## Results

### Patient and tumor characteristics

A total of 187 patients fulfilled the inclusion criteria and were included in the analytic cohort. Median age was 67 years (IQR 57–74), and 47.1% (*n* = 88) were female. Median BMI was 24.7 kg/m2, and the median age-adjusted CCI was 4 (IQR, 2–7); most patients were categorized as ASA grade ≤ 2 (*n* = 117, 62.6%). Baseline liver disease was present in one-third of patients, including steatosis (*n* = 39, 20.9%) and cirrhosis (*n* = 35, 18.7%), whereas clinical portal hypertension was documented in 2.7% (*n* = 5) of patients. Most patients underwent liver resection for malignancies, 25.7% for hepatocellular carcinoma (HCC) (*n* = 48), 24.6% for cholangiocarcinoma (CCA) (*n* = 26), and 15.5% for colorectal liver metastasis (CRLM) (*n* = 29), while benign liver and biliary diseases accounted for 23.6% of cases (*n* = 44). Overall, 33.1% (*n* = 59) and 7.9% (*n* = 14) of patients underwent anatomically major and technically major resections, respectively. Hilar lymphadenectomy was performed in 23.5% (*n* = 44) of patients, and 11.2% (*n* = 21) required biliary resection and/or reconstruction. Mean operative time was 405 min (IQR 300–500). Intermittent hilar clamping was used in 67.9% of cases (*n* = 127). The EBL was > 300 mL in 37.4% (*n* = 70), while 9.1% (*n* = 17) of patients required blood transfusion. Negative surgical margins (R0 resection) were achieved in 86.1% of patients (*n* = 161) (Table 1).

**Table 1:** Patients characteristics

Characteristic	*N* = 187
**Age** (years)	67 (57–74)
**Gender**	
Male	99 (52,9%)
Female	88 (47,1%)
**BMI** (kg/m^2^)	24.7 (22.3–27)
**ASA score**	
1–2	117 (62,6%)
3–4	70 (37,4%)
CCI	4 (2–7)
**Portal vein hypertension**	5 (2,7%)
**Liver Histology**	
Healthy	113 (60,4%)
Steatosis	39 (20,9%)
Cirrhosis	35 (18,7%)
**Disease**	
Benign disease	31 (16,6%)
Biliary tract benign disease	13 (7%)
CCC	26 (13,9%)
CRLM	29 (15,5%)
HCC	48 (25,7%)
N-CRLM	18 (9,6%)
Other primitive malignancy	2 (1,1%)
**Anatomic resection**	136 (72,7%)
**Extent of resection**	
Minor	105 (59%)
Technically major	14 (7,9%)
Anatomically major	59 (33,1%)
**Biliary tract reconstruction**	21 (11,2%)
**Hilar lymphadenectomy**	44 (23,5%)
**EBL ≥ 300** mL	70 (37,4%)
**Operative time** (min)	405 (300–500)
**Hilar clamping**	127 (67,9%)
**Intra-operative events**	
No	172 (92%)
I	15 (8%)
II-III	0 (0%)
**Peri-operative blood transfusion**	17 (9,1%)
**R0 Resection margin**	161 (86,1%)

Data are presented as median (IQR) for continuous variables and n (%) for categorical variables

BMI, body-mass index; ASA, American Society of Anesthesiology; CCC, cholangiocarcinoma; CRLM, colorectal liver metastasis; HCC, hepatocellular carcinoma; N-CRLM, non-colorectal liver metastasis; CCI, Charlson Comorbidity Index

**Fig. 1:**
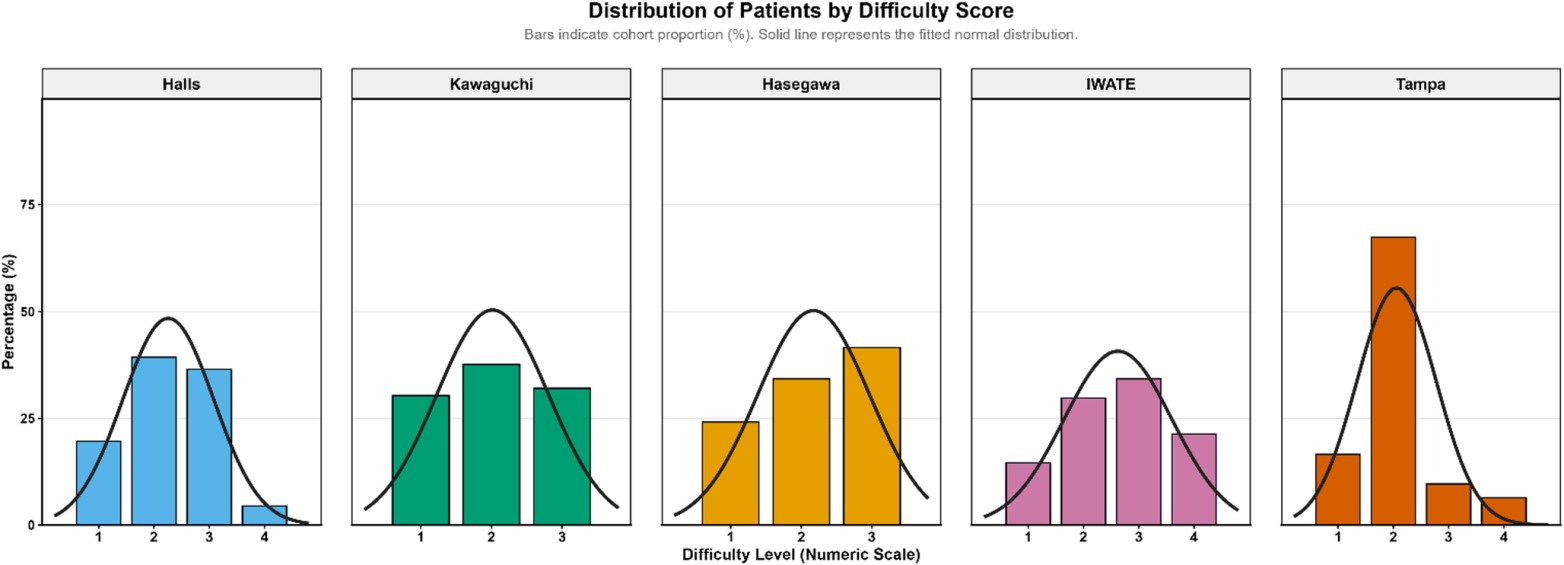
Patients distribution and stratification across the five different difficulty scoring systems

The assessment of surgical difficulty varied drastically depending on the DSS. While the Kawaguchi and Tampa DSS showed a central tendency, the Halls, IWATE, and Hasegawa scores were more skewed toward higher difficulty levels (Fig. 1). First of all, while the laparoscopic scores (IWATE, Kawaguchi, Halls, Hasegawa) were applied strictly to liver resections (*n* = 178), the Tampa score was calculated for the entire cohort (*n* = 187) including isolated biliary procedures. Among laparoscopy-based DSS, Kawaguchi showed a balanced distribution across its levels (Level I: 30.3%; Level II: 37.6%; Level III: 32.0%), while the Hasegawa and IWATE scores were skewed towards the higher difficulty categories, with 41.6% classified as “High” (Hasegawa) and 55.6% as “Advanced/Expert” (IWATE). The Halls DSS showed a central clustering, with 75.8% (*n* = 135) classified as “Moderate” or “High” complexity. Conversely, the Tampa DSS shows a selective capacity in identifying high-complexity cases, with most patients falling in the “intermediate” group (67.4%, *n* = 126), while only 6.4% were classified in the “most demanding” group (*n* = 12) (Table 2).

**Table 2:** Patients distribution based on different DSS

**Halls DSS** (*n*. 178)	
Low	35 (19,7%)
Moderate	70 (39,3%)
High	65 (36,5%)
Extremely high	8 (4,5%)
**Kawaguchi DSS** (n. 178)	
I	54 (30,3%)
II	67 (37,6%)
III	57 (32,0%)
**Hasegawa DSS** (n. 178)	
Low	43 (24,2%)
Medium	61 (34,3%)
High	74 (41,6%)
**IWATE DSS** (n. 178)	
Low	26 (14,6%)
Intermediate	53 (29,8%)
Advanced	61 (34,3%)
Expert	38 (21,3%)
**TAMPA DSS** (n. 187)	
Less Demanding	31 (16,6%)
Intermediate	126 (67,4%)
More Demanding	18 (9,6%)
Most Demanding	12 (6,4%)

### Postoperative outcomes

A total of 77 (41.1%) patients developed postoperative complications, with 14.4% (*n* = 27) of patients developing severe complications (Clavien-Dindo *≥* 3). Only 15 (8%) patients developed intraoperative adverse events, which were all grade I according to the Oslo classification, while unplanned conversion rate was 4.3% (*n* = 8). Median LOS was 6 days (IQR, 4–9), whereas 30-day readmission and reintervention rates were 6.4% (*n* = 12) and 2.7% (*n* = 5), respectively. The 90-day mortality occurred in 6 patients (3.2%). The TOLLS was achieved in 66.8% of the patients (*n* = 125), thus representing the composite ideal postoperative outcome after liver resection (Table 3).

**Table 3:** Postoperative outcomes

Characteristic	*N* = 187
**Complications**	
No	110 (58,8%)
Clavien-Dindo 1–2	50 (26,7%)
Clavien-Dindo ≥ 3	27 (14,4%)
**Unplanned conversion**	8 (4,3%)
**30-days readmission**	12 (6,4%)
**30-days reintervention**	5 (2,7%)
**90-days mortality**	6 (3,2%)
**LOS** (days)	6 (4–9)
**TOLLS**	
Achieved	125 (66,8%)
Not achieved	62 (33,2%)

LOS, length of stay; TOLLS, Textbook Outcomes in Laparoscopic Liver Surgery. *DSS comparison*

All the DSS showed a significant association between predicted difficulty and intraoperative surgical complexity. A stepwise increase in operative time and EBL was observed across the increasing predicted difficulty in all four DSS (all *p* < 0.001). Notably, the Tampa score showed the most pronounced stratification for operative time, which ranged from a median of 250 min in “less demanding” to 735 min in “most demanding”, reflecting the increased difficulty associated with advanced robotic procedures. Moreover, increasing difficulty was also significantly associated with both use and duration of hilar clamping across all DSS.

All scores correlated significantly with severe complications (*p* < 0.05). Notably, the Tampa score was the only one showing a significant increase in 90-day mortality across the levels (*p* = 0.005). Similarly, TOLLS achievement significantly decreased progressively with increasing difficulty among all DSS (*p* > 0.001), with the Tampa score showing a marked decline from 83.9% (*n* = 26) in the “less demanding” group to 16.7% (*n* = 2) in the “most demanding” group, respectively. Length of stay increased stepwise across the difficulty categories. While laparoscopy-based DSS showed a moderate increase (median of 7 days in the highest difficulty groups), the Tampa identified a subset of highly complex cases associated with significantly prolonged hospital stay. Specifically, median LOS was 9 days (IQR, 5–13) and 19 days (IQR, 10–28) in Tampa “more demanding” and “most demanding” groups, respectively (**Table Supplement 2–6**).

**Fig. 2:**
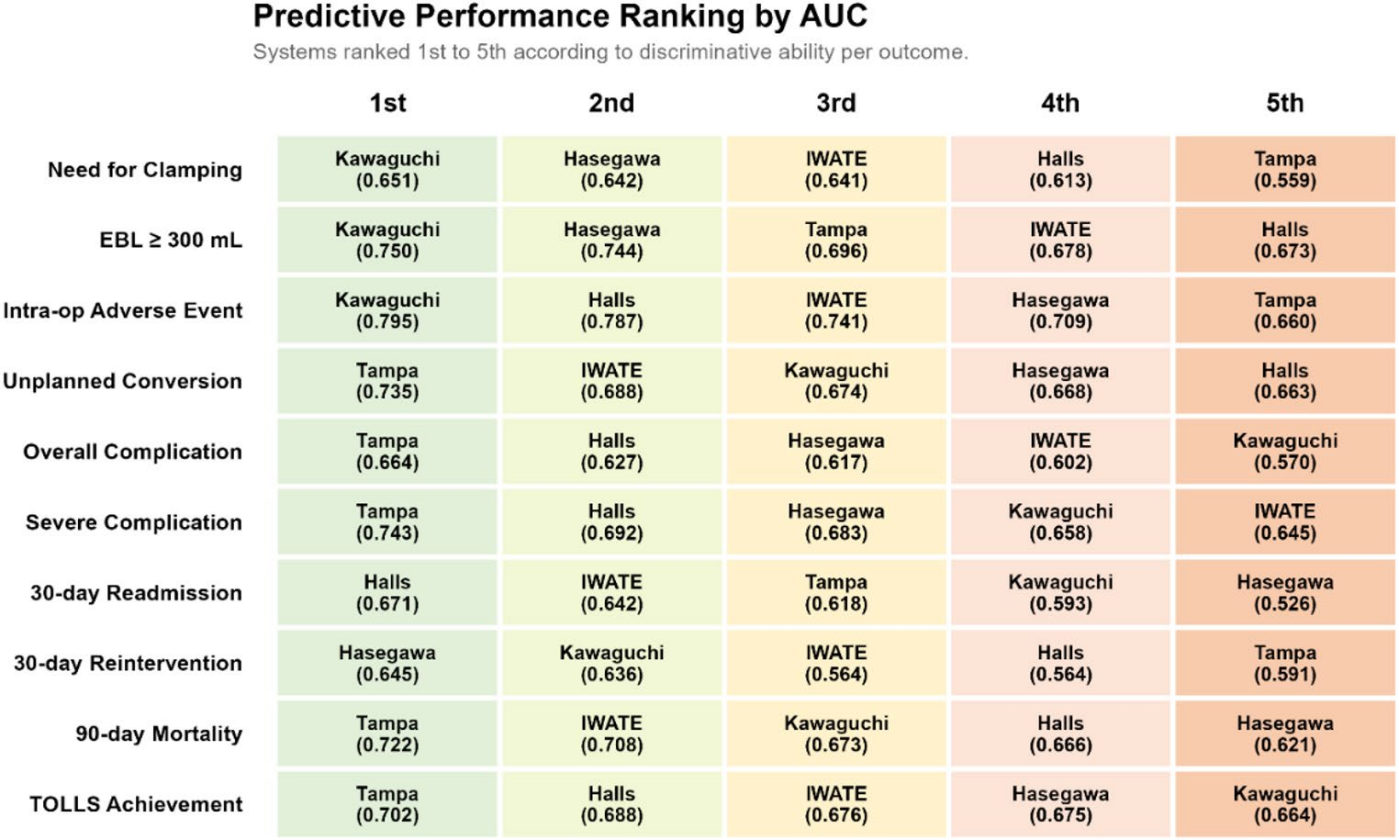
Comparative analysis of the discriminative ability among the different scoring systems in assessing intraoperative and short-term outcomes. Specific AUCs are reported in brackets

Figure 2 shows a comparison of the discriminative performance of the different DSS for various intraoperative and postoperative outcomes. On ROC-curve analysis, the Kawaguchi score appeared to be the most discriminative for assessing the need for hilar clamping (AUC 0.651), EBL exceeding 300 mL (AUC 0.750) and intraoperative adverse events (AUC 0.795). In contrast, the Tampa score outperformed the other DSS in predicting perioperative outcomes, achieving the highest AUCs for overall and severe complications (AUC 0.664 and AUC 0.743, respectively), unplanned conversion (AUC 0.735), 90-day mortality (AUC 0.722), and TOLLS achievement (AUC 0.702).

**Fig. 3:**
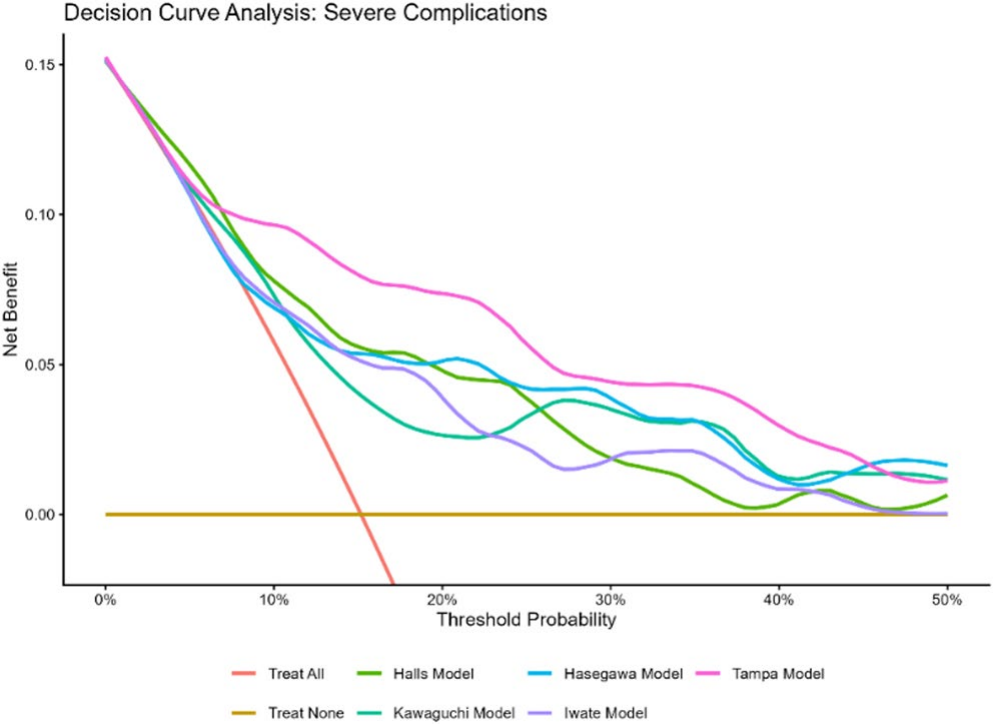
Decision Curve Analysis of adjusted models for severe complications based on different difficulty scoring systems

On multivariable analysis, the model based on the Tampa DSS was the most predictive of overall (C-index = 0.73, AIC = 220.3) and severe complication (C-index = 0.83, AIC = 128.0), 90-day mortality (C-index = 0.952, AIC = 40.6), unplanned conversion (C-index = 0.80, AIC = 68.3), 30-day reintervention (C-index = 0.77, AIC = 53.5), and TOLLS achievement (C-index = 0.75, AIC = 203.1), showing superior goodness-of-fit achieving the lowest AIC. In comparison, when assessing the need for hilar clamping, intraoperative adverse events, and increased EBL, the model based on the Kawaguchi DSS achieved the lowest AIC (205.0, 88.9, and 207.0, respectively), while the model based on the Halls DSS showed the best goodness-of-fit for 30-day readmission (AIC = 84.0). (**Table Supplement 7–16**). Furthermore, DCA confirmed that the model incorporating the Tampa DSS was clinically more meaningful and could provide the highest net benefit when assessing the development of major complications compared to the other scores (Fig. 3). In fact, on multivariable analysis the Tampa DSS showed the highest association with severe postoperative complications (OR 6.00, 95%CI 2.72-15.0; *p* < 0.001) when compared to the other scores (**Table Supplement 17**).

## Discussion

Minimally invasive liver surgery (MILS) has expanded rapidly worldwide, driven by consistent evidence demonstrating improved short-term outcomes and reduced LOS while yielding comparable oncological results compared with open approach [21]. Specifically, in selected cases, robotic liver resection has been considered equivalent in terms of clinical outcomes to laparoscopy, according to the Consensus of the European Association of Endoscopic Surgeons in 2015 [22]. Nonetheless, the implementation of the robotic platform in the field of complex hepatobiliary surgery has exposed a critical gap: most available DSS were conceived and validated in laparoscopic cohorts, raising concerns regarding their applicability in robotic-assisted surgery. While only the Ban and Iwate DSS have ever been externally validated in this subset of patients, no DSS was deemed to be superior altogether, thus their application is often a result of their ease of use in day-to-day clinical practice [1]. The current study provides a comprehensive comparative evaluation of the main DSS within a single HPB tertiary western center cohort of 187 patients. Namely, our finding suggests that, although classic DSS remain useful to estimate intraoperative technical complexity, the Tampa score encompasses superior discriminatory performance and goodness-of-fit for clinically meaningful perioperative outcomes. These results support preferential and standard use of a robotic-specific DSS when stratifying risk and planning robotic-assisted liver resections.

A fundamental distinction between the Tampa score and traditional DSS lies in their scope. In fact, laparoscopy-based DSS (IWATE, Kawaguchi, Halls, and Hasegawa) are limited to parenchymal resection and primarily rely on tumor-related and anatomical parameters. In contrast, the Tampa DSS includes also complex procedures such as biliary resection and reconstruction and lymphadenectomy, thereby reflecting the broader spectrum of procedures increasingly performed by robot-assisted surgery. In the current study, about 5% of patients underwent complex hepatobiliary procedures, not amenable to classification by “classical” DSS. This limitation is particularly relevant considering that robotic-assisted surgery is frequently selected for its technical advantages over laparoscopy in fine dissection and intracorporeal reconstruction, especially in complex procedures such as lymphadenectomy and biliary reconstruction. Therefore, the use of a DSS that does not account for this added procedural complexity may lead to suboptimal discrimination and potential misclassification.

An additional relevant observation concerns distribution across the DSS. Laparoscopy-based DSS exhibited a right-skewed pattern, assigning a large proportion of patients to the highest difficulty tiers. This likely reflects the penalization of posterosuperior segments and technically demanding locations in laparoscopy, which may be more accessible with robotic platform. The resulting “ceiling effect” reduces discriminatory granularity at the upper end of complexity. In contrast, the Tampa score preserved stratification at the extremes, identifying a distinct subset of truly high-complexity cases characterized by markedly prolonged LOS (median 19 days in the “most demanding” group). By reserving the highest category for procedures with the greatest technical and postoperative burden, the Tampa DSS offers enhanced precision for benchmarking, operative planning, and resource allocation.

The ROC curve analysis further underscored the conceptual divergence between DSS. The “classical” DSS showed stronger discrimination for surrogates variables of intraoperative complexity, such as EBL and need for hilar clamping, suggesting that parenchymal transection remains the dominant driver of intraoperative complexity and it is adequately captured by the laparoscopy-based DSS. Similarly, the Ban, IWATE, Kawaguchi, and Southampton DSS were found to be successful in predicting conversion rates and Pringle’s maneuver time in recently published literature, with the IWATE, Kawaguchi, and Southampton successfully predicting intraoperative complications and mortality [1]. Several studies that compare different DSS on patients who underwent robotic-assisted liver surgery failed to establish the superiority of any particular scoring system [21]. In contrast, the current study, demonstrated that, when evaluating clinically relevant perioperative outcomes (including severe complications, unplanned conversion, 90-day mortality, and TOLLS achievement), the Tampa score consistently demonstrated superior performance. As seen on DCA, utilizing the Tampa score to guide perioperative assessment can be the most effective way of identifying high-risk patients while minimizing false-positives.

While tumor characteristics and location greatly influence the intraoperative phase, patient characteristics can largely alter the postoperative trajectory, limiting the predictive capability of DSS based purely on tumor anatomy. Previous study found that patient characteristics such as comorbidities and BMI can influence surgical complexity and hinder recovery [21, 23]. To accurately predict outcomes and safety in robotic-assisted liver surgery, an ideal scoring systems should incorporate both tumor and liver characteristics, as well as parameters serving as proxy of the physiological reserve of the patient.

The current study should be interpreted considering several limitations. The retrospective single-center design may introduce potential selection bias, although the prospectively maintained database and relatively large sample size strengthen internal validity. Furthermore, the current study included patients who underwent surgery over a period of more than a decade, although 75.9% of cases (n = 142) were collected between 2023 and 2025, possibly presenting differences in perioperative patient management [24]. In the current series, all procedures were performed by surgeons with extensive experience in HPB surgery who had completed formal training on the da Vinci^®^ system, thereby in part mitigating the impact of the learning curve. Although the current results may be replicated in other high-volume centers with similarly trained surgeons and established robotic programs, the variability in institutional experience, case volume, and surgeons’ learning curve may affect outcomes. Further validation in larger multicentric populations is essential to ensure the reliability of the results in different settings.

In conclusion, the current study highlights the limitations of the “classical” DSS and demonstrated the superior versatility and accuracy of the Tampa DSS in the robotic-assisted surgery setting. While “classical” DSS maintain high accuracy in predicting intraoperative difficulty, the Tampa DSS more accurately and efficiently captures the multidimensional complexity of robotic procedures and is better related with perioperative course. The adoption of a more comprehensive scoring system in robotic-assisted liver resection is essential for robust benchmarking, outcome comparisons, and standardized patient risk stratification.

## Electronic Supplementary Material

Below is the link to the electronic supplementary material.

Supplementary Material 1

## Data Availability

The data that support the findings of this study are not publicly available due to privacy and ethical restrictions. Data are available from the corresponding author upon reasonable request, pending ethical approval.
