# Supplementary material for: Towards standardized assessment of surgical difficulty in robotic hepatobiliary surgery: a comparative validation study
Source: J Robot Surg. 2026 Jul 20;20(1):700. doi: 10.1007/s11701-026-03472-9 (PMC13384978; doi:10.1007/s11701-026-03472-9)
Supplement: Supplementary file 1 — Supplementary Material 1 [file 11701_2026_3472_MOESM1_ESM.docx]

**Towards Standardized Assessment of Surgical Difficulty in Robotic Hepatobiliary Surgery: A Comparative Validation Study**

Simone Conci, MD, PhD^1^; Giovanni Catalano, MD^1^; Serena Di Paolo, MD^1^; Tommaso Campagnaro, MD, PhD; Mario De Bellis, MD, PhD; Laura Alaimo, MD, PhD; Edoardo Poletto, MD; Andrea Ruzzenente, MD, PhD^1^

**Affiliation**:

^1^ Division of General and Hepatobiliary Surgery, Department of Surgery, Dentistry, Gynecology and Pediatrics, University of Verona, G.B. Rossi University Hospital, P. le L.A. Scuro 10, 37134, Verona, Italy

**Correspondence to:**

Simone Conci, MD, PhD

Division of General and Hepatobiliary Surgery,

Department of Surgical Sciences, Dentistry, Ginecology and Pediatrics,

University of Verona Medical School, G.B. Rossi University Hospital

Piazzale L.A. Scuro, 10, Verona 37134, Italy

Tel.: +39-045-8124655; Fax: +39-045-8027426; Email: [simone.conci@univr.it](mailto:simone.conci@univr.it)

**Supplementary Tables**

**Table Supplement 1**. Summary of the main difficulty scoring systems for minimally invasive liver resection.

| **Study** | **DSS** | **Variables included** | **Difficulty categories** |
| --- | --- | --- | --- |
| Wakabayashi et al., 2016^1^ | IWATE | Resection extent Tumor location Tumor size Liver function Proximity to major vessels HALS/hybrid method | Low Intermediate Advanced Expert |
| Hasegawa et al., 2017^2^ | Hasegawa | Resection extent Tumor location Obesity  Platelet count  on surgical time | Low Medium High |
| Halls et al., 2018 ^3^ | Halls | Resection extent Previous open liver resection Tumor type (malignant) Tumor size Neoadjuvant chemotherapy | Low Moderate High Extremely High |
| Kawaguchi et al., 2018^4^ | Kawaguchi / IMM | Procedure type: Group I 🡪 wedge, left lateral sectionectomy; Group II 🡪 anterolateral segmentectomy, left hepatectomy Group III 🡪 postero-superior segmentectomy, right-posterior sectionectomy, right hepatectomy, central hepatectomy, extended hepatectomy | I (Low) II (Intermediate) III (High) |
| Sucandy et al., 2024 ^5^ | Tampa | Resection extent Biliary reconstruction Tumor location Tumor size Malignancy Neoadjuvant chemotherapy | Low Intermediate High Very High |

**Table Supplement 2.** Intraoperative and short-term outcomes based on Halls DSS.

| **Characteristic** | **Halls 1**  N = 35 | **Halls 2**  N = 70 | **Halls 3**  N = 65 | **Halls 4**  N = 8 | ***p values*** |
| --- | --- | --- | --- | --- | --- |
| Operative Time, min. | 270 (188-376) | 360 (265-455) | 480 (378-566) | 560 (463-658) | **< 0,001** |
| EBL, mL | 100 (50-200) | 200 (100-300) | 300 (150-450) | 450 (225-675) | **< 0,001** |
| EBL > 300 mL | 6 (17,1 %) | 24 (34,3 %) | 33 (50,8 %) | 7 (87,5 %) | **< 0,001** |
| Unplanned Conversion | 0 (0 %) | 3 (4,3 %) | 4 (6,2 %) | 1 (12,5 %) | 0,352 |
| Clamping | 17 (48,6 %) | 54 (77,1 %) | 49 (75,4 %) | 7 (87,5 %) | **0,009** |
| Clamping total duration, min | 5 (0-31) | 30 (7-52) | 35 (13- 8) | 60 (33-86) | **0,003** |
| Perioperative death | 0 (0 %) | 0 (0 %) | 0 (0 %) | 0 (0 %) | - |
| Intraoperative adverse events | 0 (0 %) | 2 (2,9 %) | 10 (15,4 %) | 3 (37,5 %) | **< 0,001** |
| Post-operative complications | 9 (25,7 %) | 23 (32,9 %) | 37 (56,9 %) | 3 (37,5 %) | **0,007** |
| Severe complications | 1 (2,9 %) | 6 (8,6 %) | 20 (30,8 %) | 0 (0 %) | **< 0,001** |
| 30-days readmission | 1 (2,9 %) | 2 (2,9 %) | 7 (10,8 %) | 1 (12,5 %) | 0,178 |
| 30-days reintervention | 1 (2,9 %) | 1 (1,4 %) | 3 (4,6 %) | 0 (0 %) | 0,683 |
| 90-days mortality | 1 (2,9 %) | 0 (0 %) | 5 (7,7 %) | 0 (0 %) | 0,091 |
| Length of hospital stay, days | 4,5 (3-6) | 5 (3-7) | 7 (3,5-10,5) | 7 (2-12) | **0,003** |
| TOLLS | 31 (88,6 %) | 51 (72,9 %) | 33 (50,8 %) | 3 (37,5 %) | **< 0,001** |

Abbreviations: EBL, estimated blood loss; TOLLS, Textbook Outcomes in Laparoscopic Liver Surgery.

**Table Supplement 3.** Intraoperative and short-term outcomes based on Kawaguchi DSS.

| **Characteristic** | **Kawaguchi 1**  N = 54 | **Kawaguchi 2**  N = 67 | **Kawaguchi 3**  N = 57 | ***p values*** |
| --- | --- | --- | --- | --- |
| Operative Time, min | 315 (215-415) | 360 (258-460) | 495 (402-588) | **< 0,001** |
| EBL, mL | 100 (25-175) | 200 (100-300) | 400 (213-588) | **< 0,001** |
| EBL > 300 mL | 9 (16,7 %) | 20 (29,9 %) | 41 (71,9 %) | **< 0,001** |
| Unplanned Conversion | 0 (0 %) | 4 (6 %) | 4 (7 %) | 0,155 |
| Clamping | 33 (61,1 %) | 43 (64,2 %) | 51 (89,5 %) | **0,001** |
| Clamping total duration, min | 20 (0-42) | 30 (7,5-52,5) | 45 (25-65) | **< 0,001** |
| Perioperative death | 0 (0 %) | 0 (0 %) | 0 (0 %) | - |
| Intraoperative adverse events | 0 (0 %) | 3 (4,5 %) | 12 (21,1 %) | **< 0,001** |
| Post-operative complications | 16 (29,6 %) | 30 (44,8 %) | 26 (45,6 %) | 0,151 |
| Severe complications | 3 (5,6 %) | 10 (14,9 %) | 14 (24,6 %) | **0,020** |
| 30-days readmission | 1 (1,9 %) | 6 (9 %) | 4 (7 %) | 0,259 |
| 30-days reintervention | 1 (1,9 %) | 1 (1,5 %) | 3 (5,3 %) | 0,394 |
| 90-days mortality | 1 (1,9 %) | 1 (1,5 %) | 4 (7 %) | 0,179 |
| Length of hospital stay, days | 4 (3-5) | 6 (3-9) | 7 (4-10) | **0,001** |
| TOLLS | 45 (83,3 %) | 45 (67,2 %) | 28 (49,1 %) | **0,001** |

Abbreviations: EBL, estimated blood loss; TOLLS, Textbook Outcomes in Laparoscopic Liver Surgery.

**Table Supplement 4**. Intraoperative and short-term outcomes based on Hasegawa DSS.

| **Characteristic** | **Hasegawa 1**  N = 43 | **Hasegawa 2**  N = 61 | **Hasegawa 3**  N = 74 | ***p values*** |
| --- | --- | --- | --- | --- |
| Operative Time, min | 315 (195-435) | 345 (278-428) | 505 (387-633) | **< 0,001** |
| EBL, mL | 100 (25-175) | 200 (100-300) | 300 (150-450) | **< 0,001** |
| EBL > 300 mL | 6 (14 %) | 16 (26,2 %) | 48 (64,9 %) | **< 0,001** |
| Unplanned Conversion | 1 (2,3 %) | 1 (1,6 %) | 6 (8,1 %) | 0,144 |
| Clamping | 25 (58,1 %) | 40 (65,6 %) | 62 (83,8 %) | **0,006** |
| Clamping total duration, min | 16 (1-31) | 30 (7,5-52,5) | 45 (28,5-61,5) | **< 0,001** |
| Perioperative death | 0 (0 %) | 0 (0 %) | 0 (0 %) | - |
| Intraoperative adverse events | 0 (0 %) | 4 (6,6 %) | 11 (14,9 %) | **0,017** |
| Post-operative complications | 12 (27,9 %) | 21 (34,4 %) | 39 (52,7 %) | **0,015** |
| Severe complications | 1 (2,3 %) | 8 (13,1 %) | 18 (24,3 %) | **0,005** |
| 30-days readmission | 1 (2,3 %) | 6 (9,8 %) | 4 (5,4 %) | 0,275 |
| 30-days reintervention | 0 (0 %) | 2 (3,3 %) | 3 (4,1 %) | 0,425 |
| 90-days mortality | 1 (2,3 %) | 1 (1,6 %) | 4 (5,4 %) | 0,439 |
| Length of hospital stay, days | 4 (3-5) | 5 (3-7) | 7 (2-12) | **< 0,001** |
| TOLLS | 38 (88,4 %) | 42 (68,9 %) | 38 (51,4 %) | **< 0,001** |

Abbreviations: EBL, estimated blood loss; TOLLS, Textbook Outcomes in Laparoscopic Liver Surgery.

**Table Supplement 5**. Intraoperative and short-term outcomes based on IWATE DSS.

| **Characteristic** | **IWATE 1**  N = 26 | **IWATE 2**  N = 53 | **IWATE 3**  N = 61 | **IWATE 4**  N = 38 | ***p values*** |
| --- | --- | --- | --- | --- | --- |
| Operative Time, min | 327,5 (210-500) | 330 (273-386) | 425 (332-512) | 530 (410-650) | **< 0,001** |
| EBL, mL | 100 (25-175) | 100 (0-250) | 200 (94-307) | 300 (100-500) | **< 0,001** |
| EBL > 300 mL | 5 (19,2 %) | 14 (26,4 %) | 27 (44,3 %) | 24 (63,2 %) | **< 0,001** |
| Unplanned conversion | 0 (0 %) | 1 (1,9 %) | 4 (6,6 %) | 3 (7,9 %) | 0,297 |
| Clamping | 15 (57,7 %) | 32 (60,4 %) | 48 (78,7 %) | 32 (84,2 %) | **0,017** |
| Clamping total duration, min | 15 (0-33) | 30 (7-52) | 30 (11-54) | 45 (22-68) | **< 0,001** |
| Perioperative death | 0 (0 %) | 0 (0 %) | 0 (0 %) | 0 (0 %) | - |
| Intraoperative adverse events | 0 (0 %) | 2 (3,8 %) | 5 (8,2 %) | 8 (21,1 %) | **0,008** |
| Post-operative complications | 10 (38,5 %) | 11 (20,8 %) | 33 (54,1 %) | 18 (47,4 %) | **0,003** |
| Severe complications | 2 (7,7 %) | 2 (3,8 %) | 16 (26,2 %) | 7 (18,4 %) | **0,006** |
| 30-days readmission | 0 (0 %) | 2 (3,8 %) | 6 (9,8 %) | 3 (7,9 %) | 0,279 |
| 30-days reintervention | 1 (3,8 %) | 0 (0 %) | 3 (4,9 %) | 1 (2,6 %) | 0,452 |
| 90-days mortality | 0 (0 %) | 1 (1,9 %) | 2 (3,3 %) | 3 (7,9 %) | 0,301 |
| Length of hospital stay, days | 4 (2-6) | 4 (3-5) | 7 (2-10) | 7 (3-11) | **< 0,001** |
| TOLLS | 24 (92,3 %) | 41 (77,4 %) | 33 (54,1 %) | 20 (52,6 %) | **< 0,001** |

Abbreviations: EBL, estimated blood loss; TOLLS, Textbook Outcomes in Laparoscopic Liver Surgery.

**Table Supplement 6.** Intraoperative and short-term outcomes based on Tampa DSS.

| **Characteristic** | **TAMPA 1**  N = 31 | **TAMPA 2**  N = 126 | **TAMPA 3**  N = 18 | **TAMPA 4**  N = 12 | ***p values*** |
| --- | --- | --- | --- | --- | --- |
| Operative Time, min | 250 (166-354) | 392,5 (305-474) | 575 (478-671) | 735 (692-807) | **< 0,001** |
| EBL, mL | 100 (50-150) | 200 (100-300) | 400 (250-550) | 300 (0-700) | **< 0,001** |
| EBL > 300 mL | 3 (9,7 %) | 44 (34,9 %) | 15 (83,3 %) | 8 (66,7 %) | **< 0,001** |
| Unplanned conversion | 0 (0 %) | 4 (3,2 %) | 1 (5,6 %) | 3 (25 %) | **0,002** |
| Clamping | 19 (61,3 %) | 84 (66,7 %) | 17 (94,4 %) | 7 (58,3 %) | 0,071 |
| Clamping total duration, min | 20 (5-45) | 30 (0-60) | 45 (33-56) | 30 (0-60) | 0,096 |
| Perioperative death | 0 (0 %) | 0 (0 %) | 0 (0 %) | 0 (0 %) | - |
| Intraoperative adverse events | 0 (0 %) | 10 (7,9 %) | 2 (11,1 %) | 3 (25 %) | 0,054 |
| Post-operative complications | 7 (22,6 %) | 47 (37,3 %) | 11 (61,1 %) | 12 (100 %) | **< 0,001** |
| Severe complications | 1 (3,2 %) | 12 (9,5 %) | 7 (38,9 %) | 7 (58,3 %) | **< 0,001** |
| 30-days readmission | 1 (3,2 %) | 7 (5,6 %) | 3 (16,7 %) | 1 (8,3 %) | 0,272 |
| 30-days reintervention | 1 (3,2 %) | 2 (1,6 %) | 1 (5,6 %) | 1 (8,3 %) | 0,447 |
| 90-days mortality | 1 (3,2 %) | 1 (0,8 %) | 2 (11,1 %) | 2 (16,7 %) | **0,005** |
| Length of hospital stay, days | 4 (2-5) | 6 (4-8) | 9 (5-13) | 19 (10-28) | **< 0,001** |
| TOLLS | 26 (83,9 %) | 93 (73,8 %) | 4 (22,2 %) | 2 (16,7 %) | **< 0,001** |

Abbreviations: EBL, estimated blood loss; TOLLS, Textbook Outcomes in Laparoscopic Liver Surgery.

**Table Supplement 7**. Multivariable model comparison for overall complication based on different difficulty scoring systems.

| **Predictive Model** | **AIC** | **C-Index** | **95% CI** | **Δ AIC** |
| --- | --- | --- | --- | --- |
| Tampa Model | 220.3 | 0.734 | 0.658 - 0.810 | 0.0 |
| Halls Model | 237.1 | 0.686 | 0.607 - 0.765 | 16.8 |
| Hasegawa Model | 239.4 | 0.676 | 0.595 - 0.757 | 19.1 |
| Iwate Model | 240.9 | 0.672 | 0.593 - 0.752 | 20.6 |
| Kawaguchi Model | 242.8 | 0.652 | 0.570 - 0.734 | 22.5 |

**Table Supplement 8**. Multivariable model comparison for severe complication based on different difficulty scoring systems.

| **Predictive Model** | **AIC** | **C-Index** | **95% CI** | **Δ AIC** |
| --- | --- | --- | --- | --- |
| Tampa Model | 220.3 | 0.734 | 0.658 - 0.810 | 0.0 |
| Halls Model | 237.1 | 0.686 | 0.607 - 0.765 | 16.8 |
| Hasegawa Model | 239.4 | 0.676 | 0.595 - 0.757 | 19.1 |
| Iwate Model | 240.9 | 0.672 | 0.593 - 0.752 | 20.6 |
| Kawaguchi Model | 242.8 | 0.652 | 0.570 - 0.734 | 22.5 |

**Table Supplement 9.** Multivariable model comparison for 90-day mortality based on different difficulty scoring systems.

| **Predictive Model** | **AIC** | **C-Index** | **95% CI** | **Δ AIC** |
| --- | --- | --- | --- | --- |
| Tampa Model | 40.6 | 0.952 | 0.889 - 1.000 | 0.0 |
| Halls Model | 44.8 | 0.922 | 0.826 - 1.000 | 4.2 |
| Hasegawa Model | 46.2 | 0.922 | 0.834 - 1.000 | 5.6 |
| Iwate Model | 46.7 | 0.927 | 0.861 - 0.993 | 6.1 |
| Kawaguchi Model | 47.9 | 0.910 | 0.788 - 1.000 | 7.3 |

**Table Supplement 10.** Multivariable model comparison for unplanned conversion based on different difficulty scoring systems.

| **Predictive Model** | **AIC** | **C-Index** | **95% CI** | **Δ AIC** |
| --- | --- | --- | --- | --- |
| Tampa Model | 68.3 | 0.801 | 0.647 - 0.955 | 0.0 |
| Halls Model | 73.9 | 0.701 | 0.528 - 0.873 | 5.6 |
| Hasegawa Model | 74.0 | 0.701 | 0.555 - 0.847 | 5.7 |
| Iwate Model | 74.1 | 0.681 | 0.490 - 0.872 | 5.8 |
| Kawaguchi Model | 74.2 | 0.692 | 0.491 - 0.893 | 5.9 |

**Table Supplement 11.** Multivariable model comparison for 30-day reintervention based on different difficulty scoring systems.

| **Predictive Model** | **AIC** | **C-Index** | **95% CI** | **Δ AIC** |
| --- | --- | --- | --- | --- |
| Tampa Model | 53.5 | 0.771 | 0.584 - 0.958 | 0.0 |
| Halls Model | 53.6 | 0.813 | 0.637 - 0.988 | 0.1 |
| Hasegawa Model | 53.6 | 0.797 | 0.590 - 1.000 | 0.1 |
| Iwate Model | 54.5 | 0.758 | 0.560 - 0.956 | 1.0 |
| Kawaguchi Model | 54.6 | 0.753 | 0.566 - 0.939 | 1.1 |

**Table Supplement 12.** Multivariable model comparison for TOLLS achievement based on different difficulty scoring systems.

| **Predictive Model** | **AIC** | **C-Index** | **95% CI** | **Δ AIC** |
| --- | --- | --- | --- | --- |
| Tampa Model | 203.1 | 0.745 | 0.665 - 0.826 | 0.0 |
| Halls Model | 215.6 | 0.718 | 0.642 - 0.795 | 12.5 |
| Hasegawa Model | 217.7 | 0.709 | 0.632 - 0.787 | 14.6 |
| Iwate Model | 218.1 | 0.715 | 0.640 - 0.790 | 15.0 |
| Kawaguchi Model | 220.0 | 0.697 | 0.618 - 0.775 | 16.9 |

**Table Supplement 13.** Multivariable model comparison for need of hilar clamping based on different difficulty scoring systems.

| **Predictive Model** | **AIC** | **C-Index** | **95% CI** | **Δ AIC** |
| --- | --- | --- | --- | --- |
| Tampa Model | 205.0 | 0.718 | 0.638 - 0.797 | 0.0 |
| Halls Model | 206.8 | 0.706 | 0.622 - 0.791 | 1.8 |
| Hasegawa Model | 207.1 | 0.711 | 0.631 - 0.792 | 2.1 |
| Iwate Model | 210.3 | 0.689 | 0.603 - 0.776 | 5.3 |
| Kawaguchi Model | 217.6 | 0.642 | 0.556 - 0.727 | 12.6 |

**Table Supplement 14.** Multivariable model comparison for intraoperative adverse events based on different difficulty scoring systems.

| **Predictive Model** | **AIC** | **C-Index** | **95% CI** | **Δ AIC** |
| --- | --- | --- | --- | --- |
| Tampa Model | 88.9 | 0.874 | 0.779 - 0.968 | 0.0 |
| Halls Model | 90.9 | 0.865 | 0.769 - 0.962 | 2.0 |
| Hasegawa Model | 97.0 | 0.830 | 0.710 - 0.950 | 8.1 |
| Iwate Model | 98.5 | 0.814 | 0.702 - 0.927 | 9.6 |
| Kawaguchi Model | 102.2 | 0.775 | 0.662 - 0.887 | 13.3 |

**Table Supplement 15.** Multivariable model comparison for increased EBL based on different difficulty scoring systems.

| **Predictive Model** | **AIC** | **C-Index** | **95% CI** | **Δ AIC** |
| --- | --- | --- | --- | --- |
| Tampa Model | 207.0 | 0.782 | 0.708 - 0.855 | 0.0 |
| Halls Model | 210.0 | 0.774 | 0.703 - 0.846 | 3.0 |
| Hasegawa Model | 218.8 | 0.739 | 0.663 - 0.815 | 11.8 |
| Iwate Model | 226.9 | 0.700 | 0.623 - 0.778 | 19.9 |
| Kawaguchi Model | 228.6 | 0.713 | 0.635 - 0.790 | 21.6 |

**Table Supplement 16.** Multivariable model comparison for 30-day readmission based on different difficulty scoring systems.

| **Predictive Model** | **AIC** | **C-Index** | **95% CI** | **Δ AIC** |
| --- | --- | --- | --- | --- |
| Tampa Model | 84.0 | 0.780 | 0.605 - 0.955 | 0.0 |
| Halls Model | 84.5 | 0.781 | 0.636 - 0.926 | 0.5 |
| Hasegawa Model | 85.5 | 0.752 | 0.596 - 0.909 | 1.5 |
| Iwate Model | 86.5 | 0.756 | 0.605 - 0.906 | 2.5 |
| Kawaguchi Model | 87.6 | 0.736 | 0.590 - 0.882 | 3.6 |

**Table Supplement 17.** Results of the multivariable logistic regression analysis of the association of different difficulty scoring systems and severe complications

| **Characteristic** | **Tampa Model** | | **Halls Model** | | **Kawaguchi Model** | | **Hasegawa Model** | | **Iwate Model** | |
| --- | --- | --- | --- | --- | --- | --- | --- | --- | --- | --- |
|  | **OR** **(95% CI)** | **p-value** | **OR** **(95% CI)** | **p-value** | **OR** **(95% CI)** | **p-value** | **OR** **(95% CI)** | **p-value** | **OR** **(95% CI)** | **p-value** |
| Age | 1.06 (1.01 to 1.12) | **0.021** | 1.07 (1.02 to 1.13) | **0.003** | 1.06 (1.02 to 1.12) | **0.007** | 1.06 (1.02 to 1.12) | **0.007** | 1.06 (1.01 to 1.12) | **0.010** |
| BMI | 1.02 (0.92 to 1.14) | 0.656 | 1.05 (0.94 to 1.16) | 0.350 | 1.03 (0.93 to 1.14) | 0.537 | 1.01 (0.91 to 1.11) | 0.834 | 1.02 (0.92 to 1.12) | 0.726 |
| Female Sex | 2.53 (0.92 to 7.33) | 0.071 | 1.84 (0.72 to 4.80) | 0.201 | 1.79 (0.71 to 4.60) | 0.213 | 1.75 (0.69 to 4.49) | 0.235 | 1.79 (0.72 to 4.52) | 0.208 |
| Charlson Comorbidity Index | 0.91 (0.74 to 1.11) | 0.376 | 0.90 (0.73 to 1.08) | 0.256 | 0.93 (0.77 to 1.11) | 0.433 | 0.91 (0.75 to 1.09) | 0.325 | 0.93 (0.77 to 1.12) | 0.467 |
| Liver Histology |  | 0.208 |  | 0.121 |  | 0.276 |  | 0.279 |  | 0.342 |
| Healthy | — |  | — |  | — |  | — |  | — |  |
| Steatosis | 0.90 (0.21 to 3.43) |  | 0.29 (0.07 to 1.00) |  | 0.38 (0.09 to 1.26) |  | 0.38 (0.09 to 1.26) |  | 0.43 (0.11 to 1.39) |  |
| Sirrhosis | 2.97 (0.80 to 11.2) |  | 0.97 (0.29 to 2.96) |  | 0.91 (0.27 to 2.78) |  | 0.92 (0.27 to 2.82) |  | 1.01 (0.31 to 3.02) |  |
| Resection Extent |  | 0.234 |  | 0.345 |  | 0.780 |  | 0.800 |  | 0.516 |
| Minor | — |  | — |  | — |  | — |  | — |  |
| Technically major | 1.26 (0.25 to 5.57) |  | 2.54 (0.56 to 10.2) |  | 1.72 (0.35 to 7.81) |  | 1.66 (0.35 to 7.03) |  | 2.38 (0.49 to 10.4) |  |
| Anatomically major | 0.40 (0.09 to 1.43) |  | 0.84 (0.27 to 2.57) |  | 1.26 (0.44 to 3.67) |  | 1.14 (0.41 to 3.23) |  | 1.50 (0.52 to 4.40) |  |
| Tampa Difficulty Score | 6.00 (2.72 to 15.0) | **<0.001** |  |  |  |  |  |  |  |  |
| Halls Difficulty Score |  |  | 3.00 (1.48 to 6.56) | **0.002** |  |  |  |  |  |  |
| Kawaguchi Difficulty Score |  |  |  |  | 2.06 (1.02 to 4.28) | **0.043** |  |  |  |  |
| Hasegawa Difficulty Score |  |  |  |  |  |  | 2.73 (1.30 to 6.27) | **0.007** |  |  |
| Iwate Difficulty Score |  |  |  |  |  |  |  |  | 1.47 (0.85 to 2.59) | 0.168 |
| Abbreviations: CI; Confidence Interval; OR; Odds Ratio; BMI, body-mass index. | | | | | | | | | | |
